# Toxicity beyond the Lung: Connecting PM_2.5_, Inflammation, and Diabetes

**DOI:** 10.1289/ehp.122-A29

**Published:** 2014-01-01

**Authors:** Carol Potera

**Affiliations:** Carol Potera, based in Montana, has written for *EHP* since 1996. She also writes for *Microbe*, *Genetic Engineering News*, and the *American Journal of Nursing*.

Exposure to fine particulate matter (PM_2.5_) has been associated with increased risk of heart disease,^1^ insulin resistance (IR),^2^ and diabetes,^3^ all conditions that are characterized by inflammation.^4^ Experimental data suggest a high-fat diet^2^ may exacerbate the health effects of inhaled PM_2.5_; obese people also appear to be at increased risk.^1^ In this issue of *EHP*, investigators tease out some of the complex cellular mechanisms that could explain how PM_2.5_ may work with a high-fat diet to cause IR.^5^

Some of the authors previously reported that mice breathing PM_2.5_ and eating a high-fat diet developed IR, systemic inflammation, and increased abdominal fat, compared with mice eating the same diet but breathing filtered air.^2^ For the current study, the investigators focused on CCR2, a protein that recruits innate immune cells to insulin-sensitive tissues such as visceral fat and the liver, where it induces the inflammation characteristically seen in animal models of obesity and type 2 diabetes.^6^ They compared wild-type mice that produce CCR2 with “knockout” (CCR2^–/–^) mice that don’t. All the mice were fed a high-fat diet and then for 17 weeks were exposed to either filtered air or air containing 117 µg/m^3^ of PM_2.5_.

Among the key findings, PM_2.5_ exposure was associated with increased IR and increased levels of liver lipids in the wild-type mice. The elevated liver lipids resulted from a rise in SREBP-1c activity^5^; this protein helps regulate fatty acid synthesis.^7^ In contrast, liver lipid levels and SREBP-1c activity in CCR2^–/–^ mice were equivalent whether the mice were exposed to PM_2.5_ or breathing filtered air.^5^

IR is also characterized by abnormal insulin signaling through the AKT pathway. Reduced phosphorylation of this enzyme is associated with inflammation.^8^ The researchers found that phosphorylation of AKT was reduced in wild-type mice exposed to PM_2.5_ but unchanged in CCR2^–/–^ mice. PM_2.5_ exposure also was associated with higher levels of inflammatory F4/80 macrophages in visceral fat stores, but only in wild-type mice.^5^

The important message in all these findings is that PM_2.5_ recruits inflammatory cells via CCR2-dependent mechanisms. “This mechanism directly ties a known inflammatory mechanism in the pathogenesis of type 2 diabetes to exposure to environmental air pollution,” says study leader Sanjay Rajagopalan, division head for cardiology at the University of Maryland Medical Center.

**Figure:**
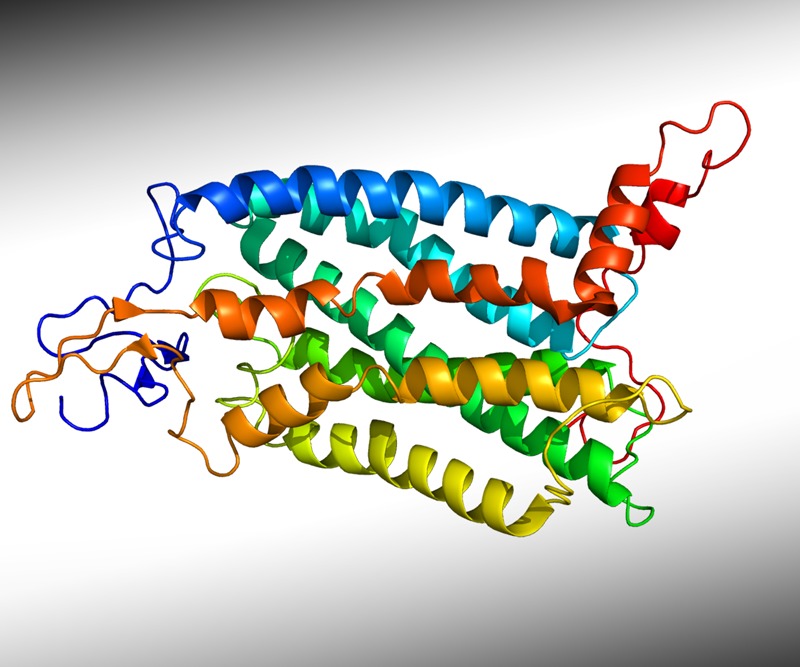
The protein CCR2 is involved in the inflammation that characterizes animal models of obesity and type 2 diabetes. Pleiotrope/Public Domain

Rajagopalan says the inflammatory damage likely creates a vicious cycle that can also contribute to cardiovascular disease and obesity. “We should look more closely at these kinds of associations in human epidemiological studies,” he says.

PM_2.5_ is generated by vehicle exhaust, burning wood and coal, and industrial processes.^9^ Annual PM_2.5_ levels in cities in China, India, and Latin America can average 100–150 µg/m^3^, comparable to the PM_2.5_ exposure in this study.^2^

“It’s long been suspected that the immune system played a major role in ‘carrying’ the toxicity of air pollutants beyond the lung,” says Matthew Campen, an associate professor at the University of New Mexico College of Pharmacy, who was not involved in the study. The results by Rajagopalan’s team strongly support the role of the innate immune system in mediating PM_2.5_ toxicity in tissues far from the lung.

The results, Campen says, also suggest that PM_2.5_ pollution could worsen cardiometabolic syndromes brought on by an unhealthy diet and lifestyle. “This public health burden may be offset by anti-inflammatory drugs or healthy diets,” he says. Alternatively, Rajagopalan proposes, “A practical solution would be to lower levels of PM_2.5_.” As Campen wryly points out, “These differing approaches may reflect the backgrounds of an environmental health scientist who understands the challenges entailed in further reductions in air pollution levels, compared with a cardiologist who understands how difficult it is to change human behavior.”
